# 756 Effects of Bioengineered Allogeneic Cellular Constructs on Macrophage Phenotype in the Context of Burns

**DOI:** 10.1093/jbcr/irad045.231

**Published:** 2023-05-15

**Authors:** Beatriz Hernaez-Estrada, Lindsay Steele, Kara Spiller

**Affiliations:** Drexel University, Philadelphia, Pennsylvania; Drexel University, Philadelphia, Pennsylvania; Drexel University, Philadelphia, Pennsylvania; Drexel University, Philadelphia, Pennsylvania; Drexel University, Philadelphia, Pennsylvania; Drexel University, Philadelphia, Pennsylvania; Drexel University, Philadelphia, Pennsylvania; Drexel University, Philadelphia, Pennsylvania; Drexel University, Philadelphia, Pennsylvania

## Abstract

**Introduction:**

The persistent inflammatory and stress responses generate in burn patients have a strong effect on the immune system. Macrophages that are activated in response to burn injuries polarize towards an immunosuppressive phenotype commonly known as M2b. Furthermore, due to sepsis, the chronic exposure to the pro-inflammatory environment also leads to an exhausted macrophage phenotype. Together these effects compromise macrophage behavior that influences the healing outcome. In clinical trials, bioengineered allogeneic cellular constructs (BACCs) have shown potential in promoting healing in patients with deep partial thickness burns. However, the mechanism(s) behind these successful effects are not totally understood yet. For this reason, the aim of this study is to investigate how the crosstalk with the BACC influence different burn-related macrophage phenotype.

**Methods:**

Primary human macrophages (N=4 healthy donors) were co-cultured with the BACC or collagen controls. Macrophages were isolated over 6 days of co-culture and their RNA was extracted for gene expression analysis, and conditioned media was collected to analyze the secretion of proteins. To assess phenotype changes, we performed gene set analysis using single sample gene set enrichment. To investigate changes in the phenotype of macrophages relevant to burn wounds macrophages were polarized to the immune suppressive M2b phenotype or LPS-exhausted phenotypes before co-culturing them with the BACC. Undifferentiated macrophages (M0) were used as controls.

**Results:**

Gene expression analysis revealed a shift in undifferentiated macrophage phenotype towards an M2 phenotype by day 3 and 6 in co-culture as demonstrated by an increase in the expression of multiple M2 genes, including MRC1 and CCL18. Secretory analysis revealed a decrease in the secretory of inflammatory proteins, like interleukin 6.

**Conclusions:**

Collectively these results demonstrate that BACCs promote the transition of macrophage phenotype towards a pro-reparative M2 phenotype which may contribute to the construct’s pro-healing capabilities.

**Applicability of Research to Practice:**

This study provides insight into the mechanism by which BACCs promote healing in the burn wound environment.

